# Attenuation of pathogenesis of *Eimeria stiedae* sporulated oocysts using Egyptian alginate propolis nanoparticles

**DOI:** 10.1186/s12917-023-03689-y

**Published:** 2023-08-18

**Authors:** Ahmed G. Hegazi, Eman E. El Shanawany, Asmaa S. El-Houssiny, Soad E. Hassan, Hassan M. Desouky, T. M. El-Metenawy, Eman H. Abdel-Rahman

**Affiliations:** 1 Zoonotic Diseases Department, Veterinary Research Institute, National Research Centre, Dokki-Giza, Egypt; 2 Parasitology and Animal Diseases Department, Veterinary Research Institute, National Research Centre, Dokki, Giza, Egypt; 3 Microwave Physics and Dielectric Department, National Research Centre, Dokki-Giza, Egypt; 4 Animal Reproduction and Artificial Insemination Department, National Research Centre, Dokki-Giza, Egypt

**Keywords:** Egyptian propolis, Alginate nanoparticles, *Eimeria stiedae*, IgG, IL-12, CD4 +, CD8 +, Immunohistochemistry

## Abstract

**Background:**

Coccidiosis is a costly and widespread infectious disease that affects mammals and causes huge losses for the global rabbit meat industry. This study evaluated the potency of Egyptian alginate propolis nanoparticles (NPs) in attenuating the infectivity of *Eimeria stiedae* sporulated oocysts. The gelification method was used to prepare alginate propolis NPs, which were then characterized using a transmission electron microscope and zeta potential analysis.

**Results:**

The results revealed that the zeta potential of the prepared alginate propolis NPs increased from − 60.60 ± 9.10 mV to –72.26 ± 6.04 mV. The sporulated oocysts were treated with 50 mg/mL of the alginate propolis NPs. Thereafter, the treated oocysts were tested for their ability to infect rabbits. The rabbits were divided into three groups: the healthy control (G1) group, the infected control (G2) group, and the treated oocyst-infected (G3) group. The rabbits were sacrificed 43 days post-infection (dpi). The infectivity of the oocysts was assessed. The treated oocyst-infected rabbits exhibited slight abdominal distension and dullness symptoms. The G3 group had no oocyst output, with a 100% reduction from 41 dpi until the end of the experiment. Immunologically, the IgG level of the G2 group gradually increased (*p* ≤ 0.05) much more than that of the G3 group. The IL-12 level in the G3 group significantly increased from 16 dpi until the end of the experiment, nearly reaching the level in healthy animals. Decreased CD4^+^ and CD8^+^ immunolabelling was observed in the liver sections of the group infected with the alginate propolis NP-treated oocysts, and there was a remarkable improvement in the histopathological parameters.

**Conclusions:**

These data indicate that Alg propolis NPs are sufficient to reduce the infectivity of *E. stiedae* oocysts.

## Introduction

Currently, Egypt has a well-established rabbit meat production industry that has a significant effect on the country’s economy [1]. According to statistics from the food and agriculture organization (FAO) statistical database, Egypt was the world’s third-largest producer of rabbit meat in 2020 [2]. The intensive production of rabbits has increased the incidence of rabbit diseases. Coccidiosis is a highly infectious disease caused by several *Eimeria* species (phylum Apicomplexa) and is one of the most serious diseases in rabbit husbandry [3, 4]. *Eimeria stiedae* is one of the most pathogenic species that infects domestic rabbits [5, 6]. *Eimeria stiedae* parasitizes bile duct epithelial cells, resulting in massive liver coccidiosis and significant economic losses [7]. Infected rabbit colonies have been reported to exhibit reduced food consumption, slow growth performance, diarrhoea, icterus, pendulous abdomen, and even high morbidity and mortality rates [8]. Furthermore, hosts infected with *Eimeria* species are susceptible to other diseases because *Eimeria* infection causes a reduction in host immunity [9]. *Eimeria* is a serious disease in rabbit farms because it is virtually difficult to eradicate [10]. Controlling this infection is crucial for improving productivity in rabbit farms [11].

Although coccidiosis is a disease that is transmitted orally from ingesting water or soil, hygiene and general management measures are critical for its control [12–16]. Usually, these measures are combined with the prophylactic or metaphylactic administration of anti-coccidial drugs [17]. The adverse effects of coccidiostats include coccidiostat resistance and even toxicity [18–20]. Long-term usage of coccidiostat in poultry feed may result in coccidiostat residues in the feed, thereby posing a health hazard [21, 22]. Various attempts have been made to control coccidiosis, but they have only had partial success[14–16, 23]. Maintaining clean sanitation breaks the *Eimeria* life cycle [24]. Inactivation or attenuation of oocysts is a more effective way to break the *Eimeria* cycle and prevent infection [25]. Thus, there is a growing demand for research into new and effective anti-*Eimeria* substances without harmful effects. Potential anti-coccidial agents, which include natural products, have been studied to reduce the risks associated with chemotherapy [16, 26–29]. Additionally, the use of natural products proves their importance in fighting against other parasitic diseases [30–38].

Propolis has recently garnered considerable interest as a potential raw natural material for developing and manufacturing innovative health-promoting medicine; it has been utilized in traditional medicine to treat various diseases since ancient times [39]. Propolis is a sticky, dark-coloured substance that bee colonies produce from plants. It has a complex chemical structure that varies depending on the source plant, geographic region, and bee species that collect it. Flavonoids, phenolic acids, and terpenoids are the main bioactive components of propolis [40–42]. Propolis is broadly used because of its biological properties, such as anti-microbial [43], immunomodulatory [44], and anti-inflammatory [45] properties. Furthermore, some clinical and experimental studies have indicated that propolis extracts have anti-parasitic properties [46, 47]. Previous studies have also indicated that propolis exhibits *in-vitro* activity against *Trypanosoma cruzi* and *Trypanosoma congolense* [48, 49], *Trichomonas vaginalis* [50], *Fasciola gigantica* [51], *Toxocara vitulorum* [52], and *Giardia duodenalis* [53]. Furthermore, clinical research suggests that propolis extracts may be effective against echinococcosis [54], schistosomiasis [55], Leishmaniasis [56], malaria [57], cryptosporidiosis [58], and toxoplasmosis [59], as well as coccidiosis in rabbits treated with zinc oxide and propolis nanoparticles (NPs) [60]. Nanoscale forms, including such NPs, have a high surface-to-volume ratio, which greatly increases the reactivity of these materials because the sample mass contains many molecules [61]. Moreover, NPs penetrate the biological barriers; protect the drug from enzyme degradation; and provide sufficient targeting, intracellular delivery, and accumulation [62, 63]. Sodium alginate is a natural anionic linear polysaccharide polymer mainly found within the cell walls of green algae, and it has been approved by the Food and Drug Administration (FDA) [64]. Alginate NPs have high bio-compatibility, non-immunogenicity, and non-toxicity. Additionally, they can be used as anti-microbial [65] and anti-toxoplasmosis [66] agents when propolis is loaded onto them.

There is limited information about the anti-coccidial characteristics of propolis in the literature. Considering the therapeutic potential of propolis and the need for new coccidiosis treatment alternatives, this study was designed to determine if Egyptian propolis extracts loaded on sodium alginate NPs can affect the infectivity of *Eimeria stiedae* sporulated oocysts.

## Materials and methods

### Sporulation of *E. stiedae* oocysts

The oocysts of *E. stiedae* were collected from naturally infected slaughtered rabbit gallbladders by bile sedimentation with extensive washing by saline to remove bile and separating oocysts. The collected oocysts were counted according to Ryley et al. [67] and identified as described by [68]. The collected oocysts were incubated for 3 d in 2.5% potassium dichromate solution at 26 °C to allow them to sporulate. The sporulated oocysts were stored at 4 °C until used.

### Propolis

The propolis sample was collected from a bee farm in Egypt’s Dakahlia governorate. The resinous materials were stored in a dark bag at 4 °C until ethanol extraction was performed. The propolis was extracted at room temperature by cutting 50 g of the sample into small pieces and adding them to 500 ml of 70% ethanol (twice after 72 h). The vacuum was used to evaporate the alcoholic extract at 50 °C until dry [65]. The phytochemical and biological analyses were previously evaluated for locally prepared propolis [69]. The extracted sample percentage was 5.1 g/dry weight. The sodium alginate and calcium chloride were of analytical grade and were supplied by ROTH, Germany, and Qualikems, India, respectively.

### Propolis alginate nanoparticle preparation 

The preparation of propolis alginate (propolis–Alg) nanoparticles (NPs) was performed using a controlled gelification method [70] based on the ionotropic gelation of polyanion with CaCl2. A 0.1% w/v concentration of alginate was dissolved in distilled water at room temperature. Next, 5 mg/ml ethanol propolis extract was mixed with the alginate solution for 24 h. After mechanical stirring, 5 ml of 36 mM CaCl2 solution was added dropwise under constant stirring to the Alg–Pomeg solution to stimulate gelification. The NP suspensions were further stirred for 3 h at room temperature. Subsequently, the prepared NPs were freeze-dried for storage.

### Transmission electron microscope

A transmission electron microscope (TEM) was used to analyse the morphology of the propolis–Alg NPs (JEM-HR-2100 microscope operated at 120 kV, Japan). A sample of the NPs’ suspension was dropped onto a copper grid. After complete drying, the sample was stained using phosphotungstic acid [65].

### Zeta potential

Dynamic light scattering (DLS) was used to determine the zeta potentials of propolis, Alg NPs, and propolis–Alg NPs (Nano-Sizer SZ90, Malvern Instruments, UK). The average value was calculated after measuring the aqueous NP suspension samples three times [65].

### Propolis alginate nanoparticles in the treatment of* E. stiedae* sporulated oocysts

*E. stiedae* sporulated oocysts were treated with 50 mg/ml of propolis–Alg NPs for 24 h [58]. The treated *E. stiedae* sporulated oocysts were washed 3 times with propolis–Alg NP extract by centrifugation at 5,000 rpm for 10 min. The oocysts were resuspended in 20 ml of deionized H_2_O and counted according to Fisher and Kelly [71] using a McMaster counting chamber and prepared for inoculation into rabbits.

### Infectivity of propolis alginate nanoparticles when treating oocysts in rabbits

White New Zealand rabbits weighing 1 kg–1.5 kg at 4–6 weeks of age were used in this study. The rabbits were purchased from the Department of Animal Production, Faculty of Agriculture, Cairo University (Rabbit Unit). Faecal examination using the floatation method was performed daily for three days before infection to confirm the absence of *E. stiedae* and other coccidian oocysts. The rabbits were conditioned for 15 days before the experiment began. All animal experimentation was conducted according to the Ethics Committee of the Medical Research of National Research centre (NRC), Egypt (approval number 1474052022). The rabbits were slaughtered under anaesthesia (xylazine 5 mg/kg), which was administered intramuscularly.

A rabbit model was used to evaluate the infectivity of *E. stiedae* oocysts treated with propolis–Alg NPs. The rabbits were divided into three groups of four and inoculated intragastrically [72] using a ball-point neonate feeding needle (24-gauge syringe, Popper and Sons, Inc.) attached to a tuberculin syringe. Group 1 (G1) was the healthy control group; Group 2 (G2) was experimentally infected with 5 × 10^4^ sporulated non-treated oocysts (i.e., the infected control group) [73]; and Group 3 (G3) was inoculated with 5 × 10^4^ treated oocysts with a 50 mg/ml concentration. All groups were kept under observation until the end of the experiment, and all rabbits were sacrificed 43 days postinfection (dpi).

### Parameters evaluated

#### Oocyst count

Individual rabbit faecal samples were obtained from the rectum and placed in small (2″ × 2″) polythene bags. The samples were collected every day from the second week postinfection (wpi) to the end of the experiment to monitor oocyst shedding. Using the McMaster technique, coccidia oocysts were counted per gram (OPG) of faeces [72]. The infectivity of the treated *E. stiedae* oocysts was assessed by comparing the mean numbers of oocysts present in groups inoculated with pretreated oocysts versus infected control rabbits. The oocyst value and reduction rate were calculated according to Lan et al. [74] as follows:.

- Oocyst value (%) = (OPG for groups inoculated with pretreated oocyst/oocyst of control infected group)* 100.
- Reduction rate (%) = [(OPG of control infected group − OPG of groups inoculated with pretreated oocyst)/OPG of control infected] ∗ 100.

### Detection of *E. stiedae*-specific antibodies

#### Serum samples

Blood samples were collected from the ear veins of all groups of experimental rabbits and placed in sterile containers every 4 days, from zero dpi to the end of the experiment. Serum was isolated and stored at − 20 °C until further analyses could be conducted.

#### Antigen preparation

*E. stiedae* oocyst antigen was prepared according to Rose and Mocke [75]. The oocysts were homogenized for 15 min on ice, followed by sonication for 5 min. The homogenates were centrifuged at 13,000 rpm for 45 min at 4 °C. The protein content of the supernatant was determined according to Lowry [76]. The antigen was aliquoted and stored at − 20 °C until it was used.

#### *E. stiedae*-specific IgG assay

An enzyme-linked immunosorbent assay (ELISA) was used to determine the presence of *E. stiedae*-specific IgG. ELISA was performed according to Oldham [77] and El Shanawany [78, 79], with some modifications. Checkerboard titration was performed to determine the optimum concentration of antigen, conjugate and sera dilutions. The plate was coated with prepared oocyst antigen. After coating, 100 μl of diluted tested sera in dilution 1:100 were added to each well. Peroxidase-labelled antirabbit IgG (Sigma) was diluted 1: 1000 and was used. Orthophenylenediamine P-6912 0.05% (Sigma) and hydrogen peroxide 0.1% were added in 100 µl volumes per well. Absorbance was read at 450 nm on an automatic micro ELISA reader ELx 800 (BIOTEK instrument, INC, Germany). The optical density (OD) cut-off value was determined using the method of Almaza´n et al. [80]. Cut off value was subtracted from all ODs.

#### Quantification of IL-12 in rabbit sera

IL-12 concentrations in rabbits were calculated using commercially available sandwich ELISA kits obtained from the Bioneovan Co., Ltd. (Beijing, China) and performed according to the manufacturer's instructions.

#### Liver lesion scores

All rabbits were dissected at the end of the study,. All rabbit's livers were thoroughly examined for grossly evident hepatic lesions [6]. For determining the severity of hepatic coccidiosis, and the protection percentage of lesions, focal lesions of the livers were scored according to Abdel Megeed and Abu El Ezz [81].

#### Histopathological examinations

From each rabbit, G2 and G3 liver tissue specimens were rapidly removed and fixed by adding them in 10% neutral buffered formalin, then embedded in paraffin and cut into Sects. (4–5 µm) added on slides. For light microscopic examination, sections were stained using hematoxylin and eosin stain (HE) [82].

#### Immunohistochemical determination of CD 4 + and CD8 + 

Formalin-fixed liver tissues of G2 and G3 were embedded in paraffin, then cut into three different Sects. 4-μm in thick and placed on positively charged slides. The paraffin sections were deparaffinized using xylene and rehydrated through series of a graded ethanol. Antigen retrieval was accomplished by steaming the slides in suitable buffers at various temperatures. To reduce nonspecific background staining caused by endogenous peroxidase, a 3% H_2_O_2_ methanol solution was used. For immunostaining, the horseradish peroxidase amplified system, CD4 + , and CD8 + monoclonal antibodies were used (Thermo Scientific, Lab Vision Corporation, Fremont, USA). Three components were used in this system: the primary antibody specific for the antigen to be localized, the secondary antibody capable of binding both primary antibodies, and the horseradish peroxidase enzyme. Finally, the substrate/chromogen reagent diaminobenzidine (DAB) was used to visualize the reaction. The number of immunohistochemical-positive cells as the mean number of brown cells per slide was identified using the ImageJ program (NIH) version 1.49 [83].

### Statistical data analysis

Data are expressed as mean ± standard deviation (SD) of the mean. GraphPad Prism Software was used to compute statistics (version 6; GraphPad Software, Inc, La Jolla, CA, USA). The significance level of results was at the level *p* ≤ 0.05.

## Results

### Transmission electron microscopy (TEM)

**Fig. 1:**
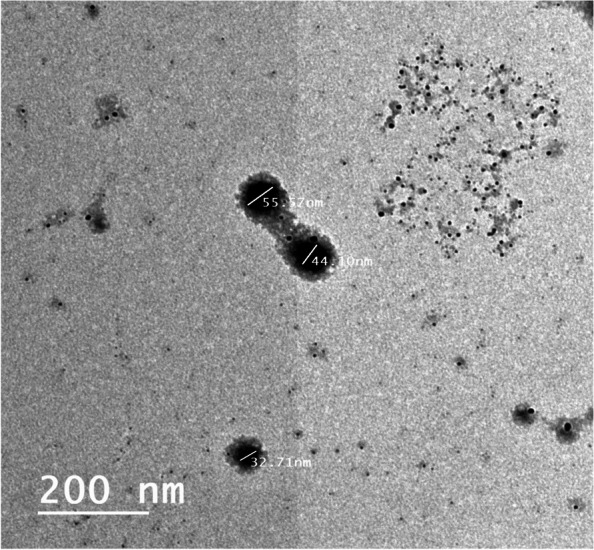
TEM image of Alginate propolis nanoparticles

The result of TEM shows that propolis-ALg NPs were distinct and spherical and had a small size nanometer range as the particle size average was 30 nm (Fig. 1).

### Zeta potential

**Fig. 2:**
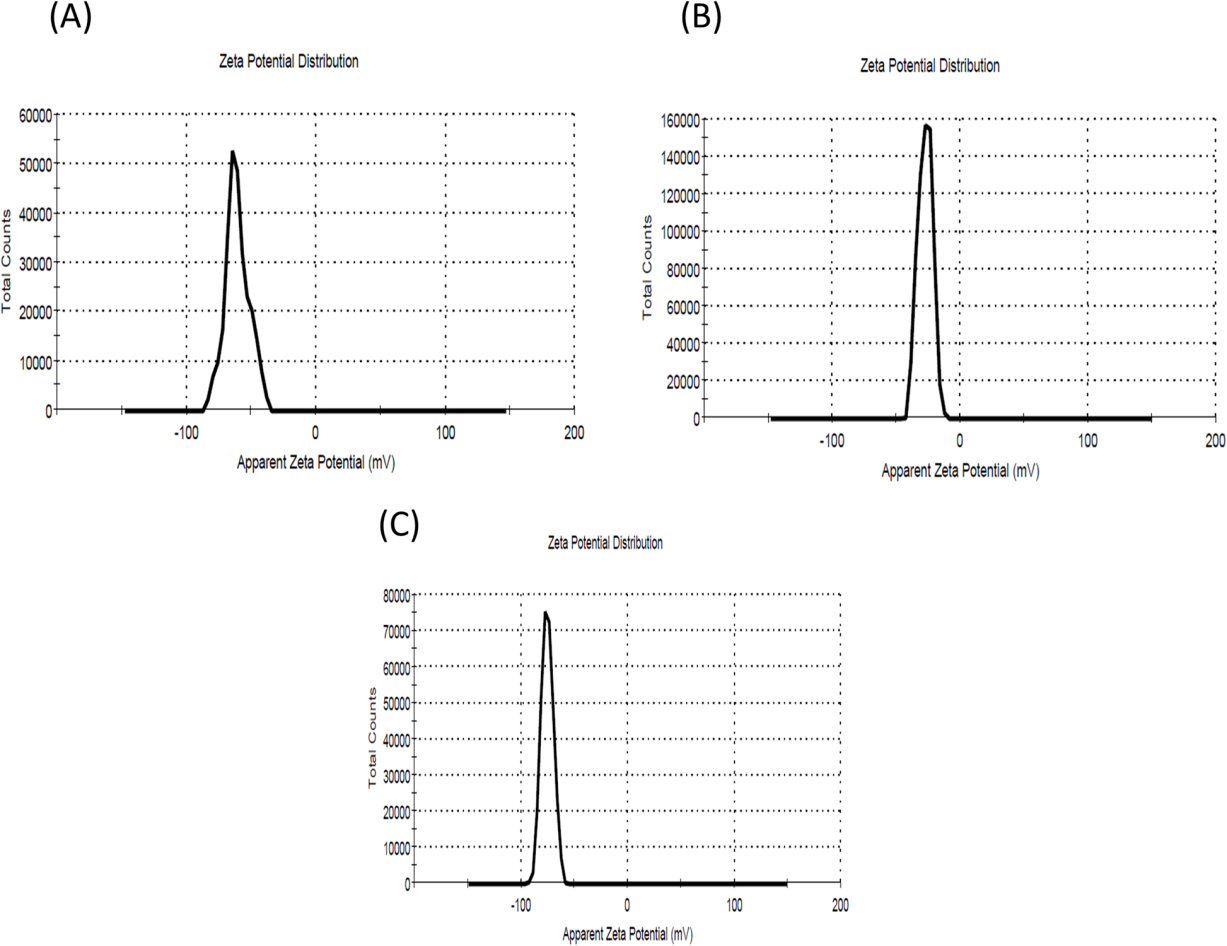
Zeta potential of Propolis (**A**), alginate nanoparticles (**B**), and alginate propolis nanoparticles (**C**)

The result of zeta potential illustrated that the zeta potential of propolis was negative with a value of -28.10 ± 5.54 mV (Fig. 2a). A negative zeta potential value was found for alginate propolis NPs (Fig. 2b). Figure 2c showed that the addition of alginate NPs to propolis results in increases in the value of zeta potential value reaching -72.26 ± 6.04 mV.

### Clinical symptoms

In the control group infected with non-treated sporulated oocysts *E. stiedae*, the rabbits presented clinical symptoms, such as looking weak, brown watery diarrhea, and loss of appetite after the second-week post-infection. Only slight abdominal distension and dullness were observed in G3group. No clinical symptoms were noted in the healthy control group.

### Oocysts per gram output

**Fig. 3:**
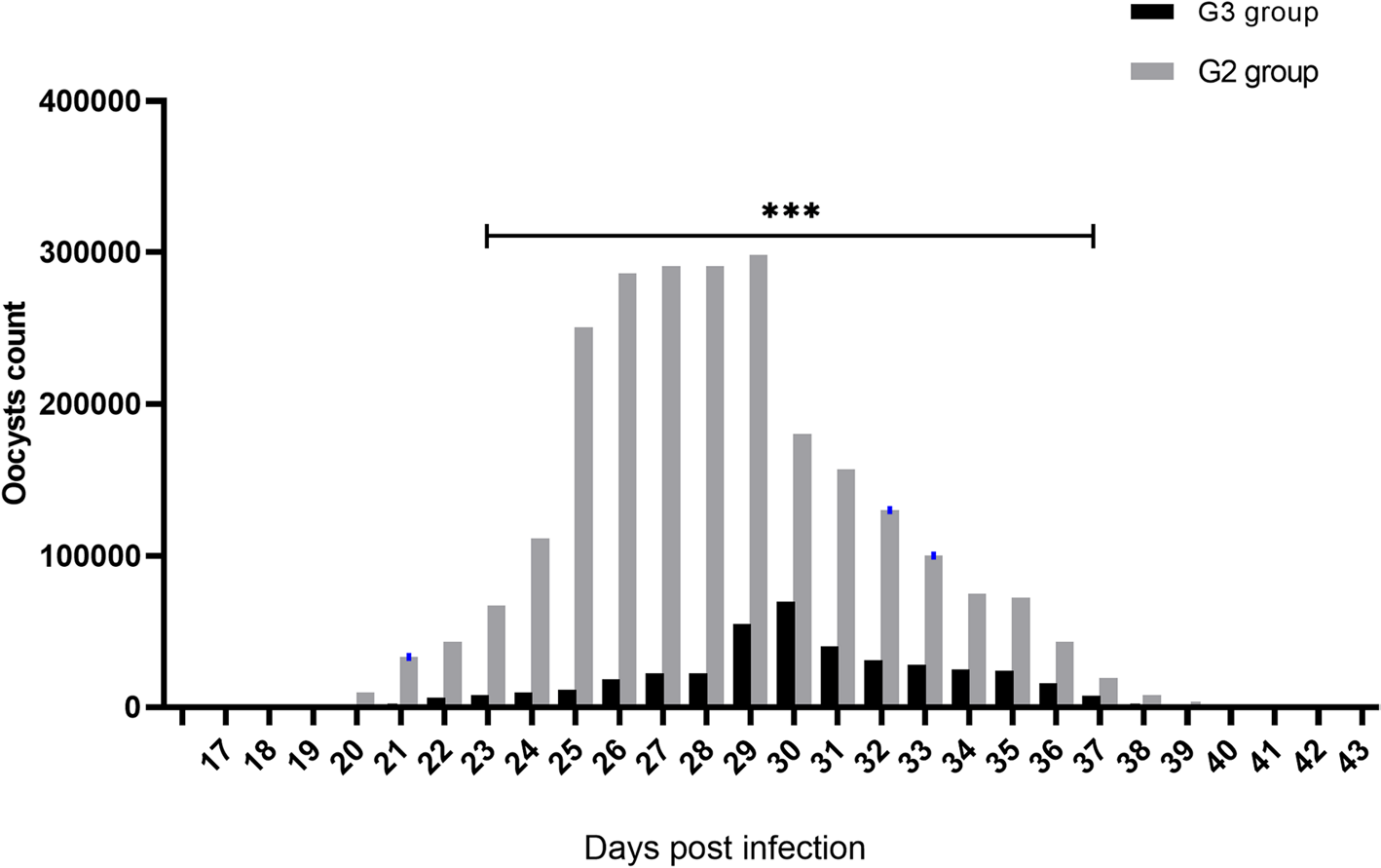
Fecal oocyst count of rabbits infected with in *vitro* treated and non-treated *E. stiedae* sporulated oocysts. Data are shown as mean ± SD. ***showed statistical significance (*p*˂0.001) between the tested groups. One-way ANOVA was used and followed by multiple comparison tests

The differences in oocyst count between G2 and G3 groups were observed in Table 1 and Fig. 3. The oocyst,s number per gram of feces was absent during the first 18 dpi in G2 group. The group infected with treated oocysts showed the first appearance of oocysts at the 19th dpi, which was significantly different (*p* < 0.001) than G2 group, with a reduction percentage of 96.83%. Fecal examination on 29th dpi revealed the highest mean number of oocyst output (298,019.8 ± 33.50) in the infected control group, which significantly (*p* < 0.001) differed from than group infected with treated oocysts (22,609.8 ± 15.90) with reduction percentage 81.57%. No oocyst output was found in the group infected with treated oocysts, with a reduction percentage of 100% from 41st dpi until the end of the experiment.

**Table 1:** Comparison of oocysts count, oocysts value (%), and oocysts reduction rate (%) between rabbits experimentally infected with in *vitro *treated and non-treated *E. stiedae *sporulated oocysts at different days post-infection

Days post infection	Rabbit groups		Reduction (%)	Oocyst value (%)
	Infected with non-treated oocysts	Infected with treated oocysts		
17	0 ± 0	0	––-	0.00
18	0 ± 0	0	––-	0.00
19	399.16 ± 5.078	0	100.00	0.00
20	9805.3 ± 10.70	310.83 ± 16.88***	96.83	3.17
21	33,133.5 ± 435.87	2586.6 ± 43.66***	92.25	7.75
22	43,303 ± 4	6201.1 ± 6.58***	85.68	14.32
23	66,996 ± 18.62	7806.6 ± 25.03***	88.35	11.65
24	111,502 ± 14.83	9810 ± 17.88***	91.20	8.80
25	250,408.6 ± 7.65	11,538.3 ± 36.56***	95.39	4.61
26	286,115 ± 18.708	18,425.3 ± 22.68***	93.56	6.44
27	291,019.8 ± 39.549	22,606.6 ± 50.46***	92.23	7.77
28	298,019.8 ± 33.50	22,609.8 ± 15.90***	92.23	7.77
29	298,007.3 ± 7.312	54,929.8 ± 18.29***	81.57	18.43
30	180,006.1 ± 6.112	69,920.3 ± 18.09***	61.16	38.84
31	157,008.3 ± 8.310	40,023 ± 19.69***	74.51	25.49
32	130,007.5 ± 8.803	31,013 ± 10.53***	76.15	23.85
33	100,006.1 ± 8.953	28,012 ± 11.50***	71.99	28.01
34	75,008 ± 9.818	25,009.3 ± 8.80***	66.66	33.34
35	72,107 ± 9.330	24,005.3 ± 4.80***	66.71	33.29
36	43,207.5 ± 8.871	15,809.8 ± 11.53***	63.41	36.59
37	19,206.3 ± 8.041	7511.6 ± 16.02***	60.89	39.11
38	8207.3 ± 7.366	2408.8 ± 7.70***	70.65	29.35
39	3607 ± 6.870	1810 ± 11.52***	49.82	50.18
40	2106 ± 7.440	204 ± 4.69***	90.31	9.69
41	1203 ± 4.490	0 ± 0	100.00	0.00
42	509.5 ± 9.934	0 ± 0	100.00	0.00
43	204.5 ± 4.96	0 ± 0	100.00	0.00

Data are expressed as Mean ± SD. *** Significant differences at *P* < 0.001

### *E. stiedae* in rabbits and parasite-specific IgG antibody levels in sera

**Fig. 4:**
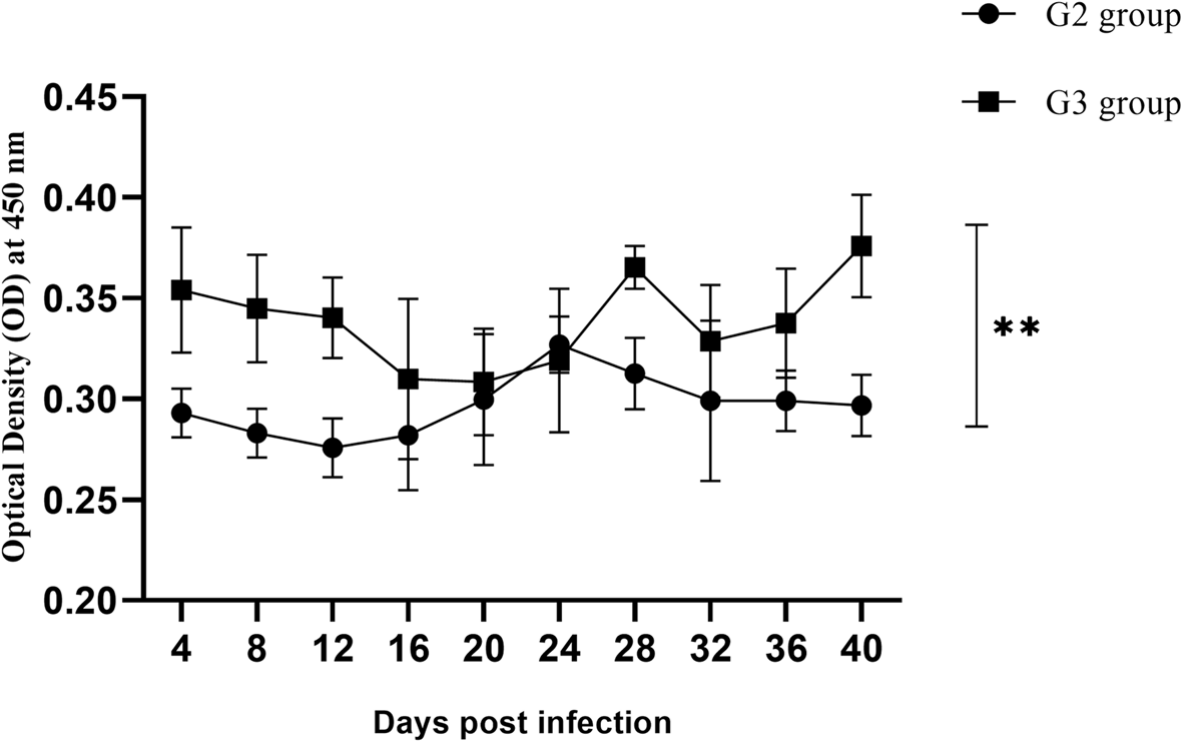
Parasite-specific IgG absorbance values in rabbits infected with alginate propolis NPs treated oocyst (G3) and control infected rabbits (G2). Data are shown as mean ± SD. *signifies significance (*p* ≤ 0.05) between the tested groups. One-way ANOVA was applied

Specific IgG levels appeared 4 days post-infection in rabbits either infected with treated or untreated oocysts. But significant differences (*p* ≤ 0.05) in IgG level were noted between both groups at 28 days post-infection, and this difference was increased until the end of the experiment. A gradual significant (*p* ≤ 0.05) increase in IgG level was observed in infected rabbits with untreated oocysts compared with the group infected with treated oocysts (Fig. 4).

### Detection of IL-12 level in rabbits

**Fig.5:**
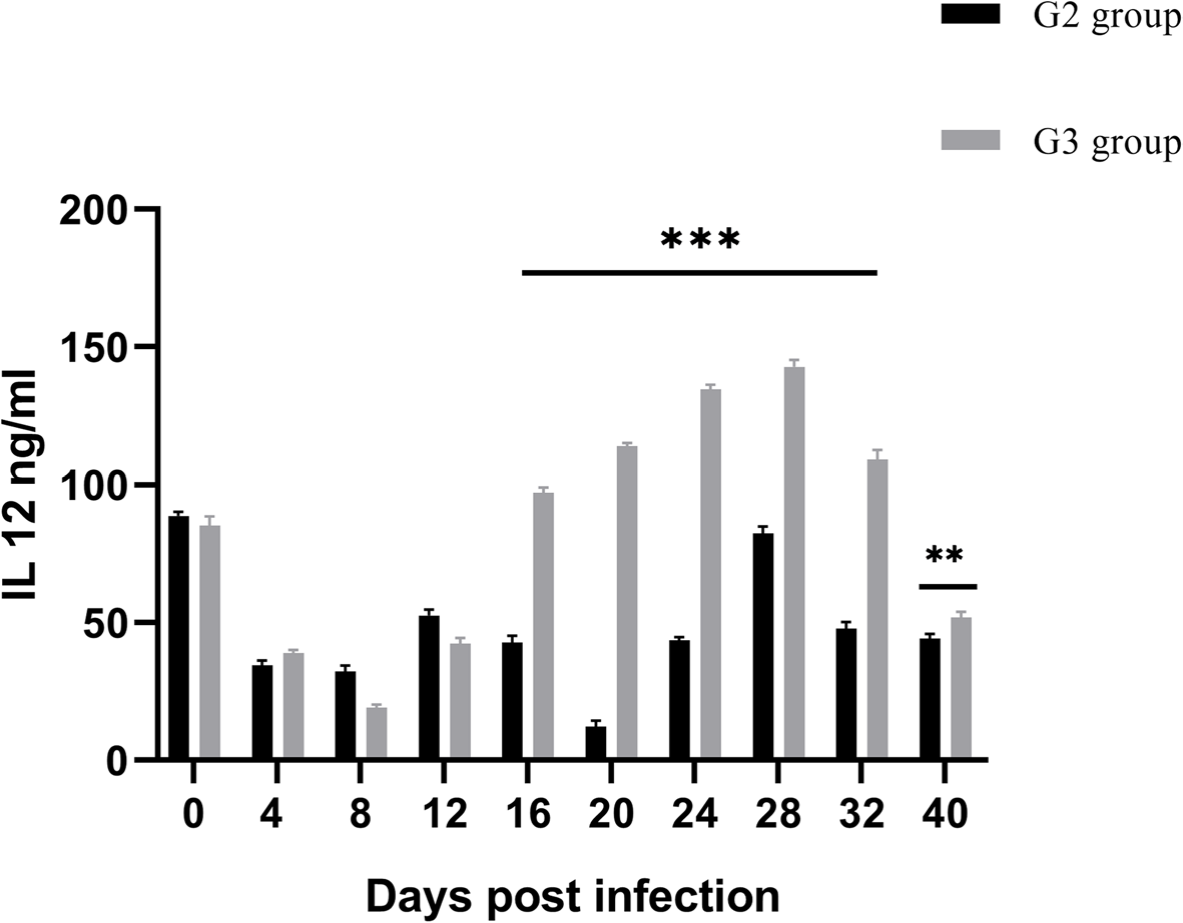
The serum levels of IL 12 in rabbits infected with ALg propolis NPs treated oocysts (G3) and control infected group (G2). The IL 12 was quantified using ELISA. Data showed as mean ± SD, * (*p* ≤ 0.05) indicates significance between tested groups. The one-way ANOVA was applied

Current results showed a significant decrease in IL-12 concentration in both groups compared with healthy animals but with no significant differences in G3group from the first of the experiment until 12 days post-infection. However, when compared to G2 group, the IL 12 concentration level increased significantly (*p* ≤ 0.05) 16 days post-infection in the group infected with treated oocysts, reaching a peak 28 days day post-infection. (Fig. 5). The IL 12 concentration level still significantly increases in G3 group to be near the healthy level recorded in rabbits before infection than control infected group until the end of the experiment.

### Liver lesion score

**Fig.6:**
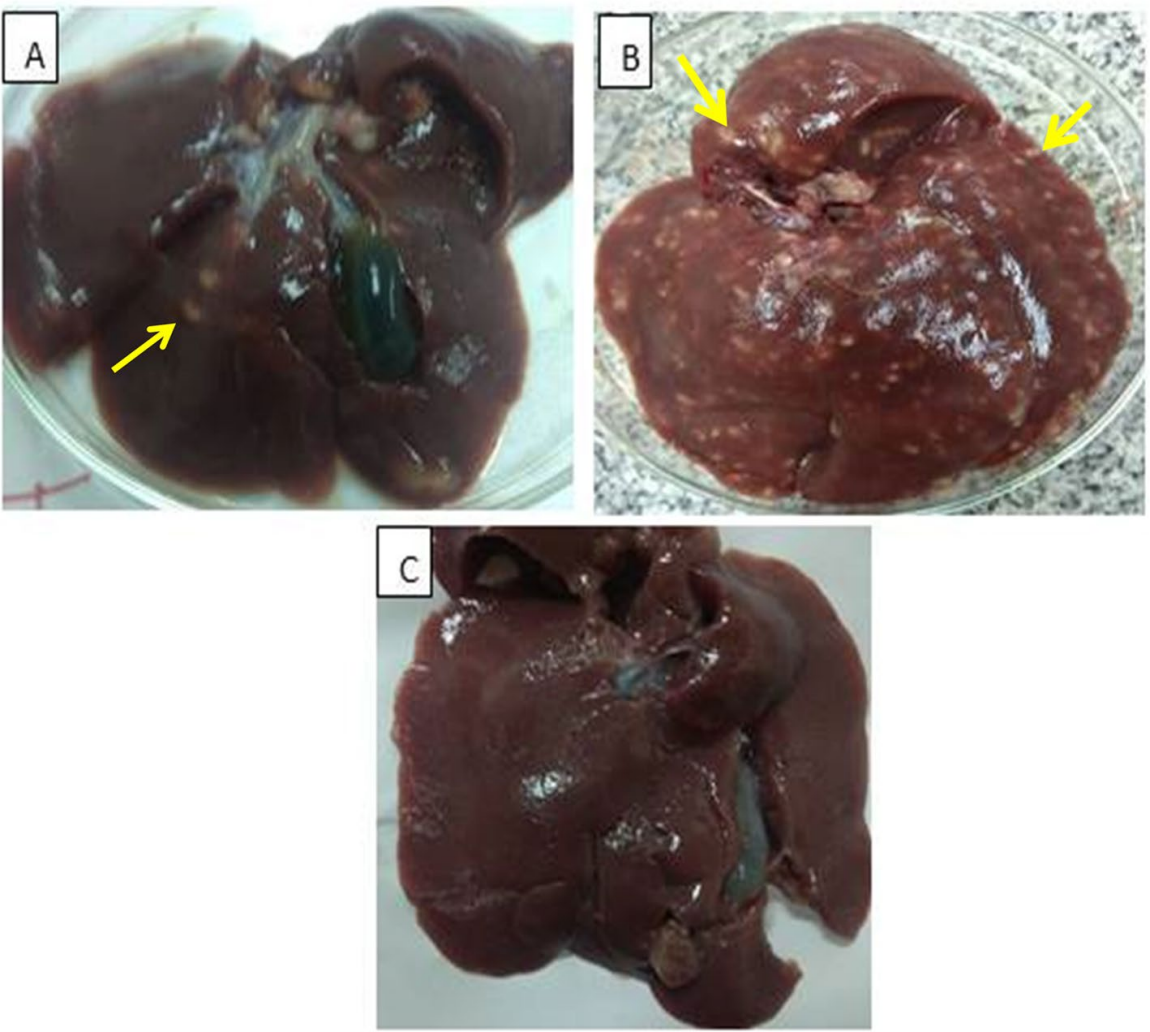
Grading scale for the rabbit's white-spotted liver's macroscopic lesions. **A** Minimal lesion: the hepatic parenchyma is dilated by a low number of creamy foci ≤ 3 mm diameter (yellow arrow). **B** Marked lesion: the hepatic parenchyma shows numerous bosselated foci with creamy white color (yellow arrow). **C** Healthy control group Liver showed no lesion in the hepatic parenchyma

The liver lesion score of the rabbit groups was studied. In the G2 group, the livers were pale and extensively enlarged with thick creamy white and numerous boss elated foci. The livers showed a score of 4 with no protection percentage found. While the G3 group showed a significant (*p* < 0.001) low number of lesions with a score of 1, and the protection percentage was 75% in comparison with the infected control group (Fig. 6).

### Histopathological findings

**Fig.7:**
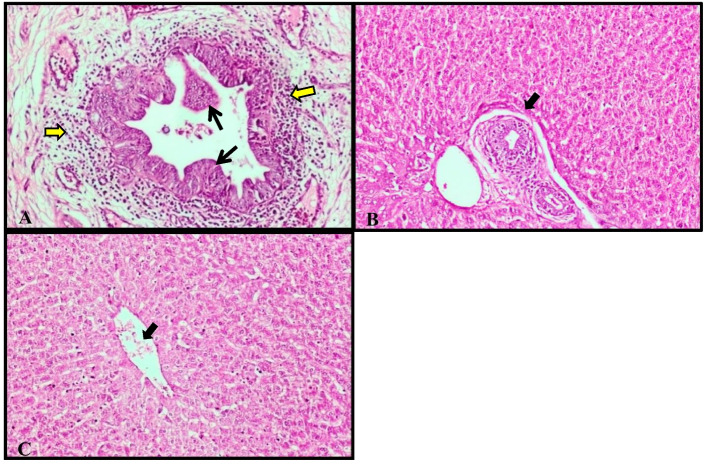
Liver of rabbit infected with *E.steadie* non–treated sporulated oocysts **A** showing proliferation, marked enlargement of bile ducts, extensive hyperplasia of the biliary epithelium forming multiple long papillary projections (arrows) (H&E, X40). **B** Showing invasion of the papillomatous proliferation of biliary epithelium with numerous and various developmental stages of the coccidian parasite (arrows) (H&E, X100). **C** Showing the cystic formation of the bile duct associated with the presence of massive numbers of oval non-sporulated oocysts (stars), and cellular debris within the lumen. (H&E,X100). **D** Higher magnification of figure C (H&E, X200). **E** Showing extensive peribiliary fibrosis (black arrow) associated with infiltration of mononuclear cells (yellow arrow), in addition to necrosis of hepatocytes (arrow head) (H&E, X100). **F** Showing multiple small scattered areas of hemorrhages in hepatic parenchyma associated with fibrosis and severe degenerative and necrotic changes of hepatocytes (yellow arrow) (H&E, X100). **G** Showing high dilatation and congestion of central vein (star) and sinusoids associated with necrosis of endothelial cells lining and centrilobular fibrous C.T. proliferation (black arrow) in addition to necrosis of hepatic cells (yellow arrow) and lymphocytic cell infiltration. (H&E, X100)

The microscopically examination of the liver of the infected group revealed severe proliferation, marked enlargement of bile ducts, extensive hyperplasia of the epithelial lining of bile ducts forming multiple fingerlike (long papillary) projections extending into the lumen of bile ducts (ductal lumina). There are numerous and diverse developmental stages of the coccidian parasite like unsporulated oocysts, and macro and micro gametocytes that can be found within the papillomatous proliferation of biliary epithelium and free in the ductal lumen. (Fig. 7A, B, C).

Moreover, the bile ducts were filled with degenerated and desquamated biliary epithelial and cellular folds debris. Severe dilatation of several bile ducts resulting in cyst formation lined with low columnar epithelium having no or minimum (papillary hyperplasia) projections into the lumens, which are filled with numerous oval non-sporulated oocysts (Fig. 7D).

Also, the infected group had a portal tract with numerous granulomas and significant intercellular fibrosis. The hyperplastic bile ducts were surrounded by loose edematous fibrous connective tissue capsule, and mononuclear inflammatory cells infiltration, mainly lymphocytes and eosinophils (Fig. 7E) that expanded portal and periportal areas and as a result, severely atrophied the neighboring hepatic parenchyma.

**Fig. 8:**
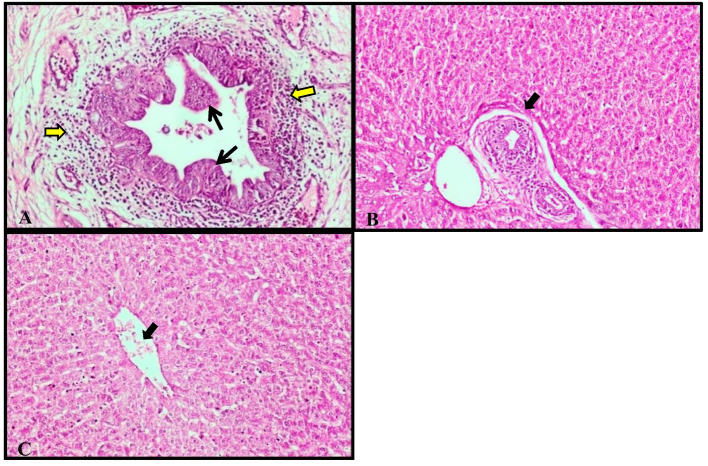
Liver of rabbit infected with *E. Steadie* alginate propolis NPs treated sporulated oocysts **A** Showing moderately dilated bile duct with mild hyperplasia of biliary epithelium (black arrows), loose edematous fibrous C.T. proliferation and mononuclear cell infiltration at the periphery of duct (yellow arrows), in the distended portal area (H&E, X100). **B** Showing a nearly normal appearance portal area and bile ducts (arrow)(H&E, X100). **C** Showing normal hepatic architecture with normal central vein and (arrow) radiating hepatic cords (H&E, X100)

In contrast, the microscopical investigation of the liver of the group infected with propolis alginate NPs treated oocysts showed remarkable improvement in the histopathological parameters in comparison with the infected untreated group. A few portal areas were moderately distended and showed a significant decrease in the number and size of coccidial bile duct granulomas with a significant reduction in collagen content deposition (fibrosis) and mononuclear cell infiltration. Moreover, decreased hyperplasia of the biliary epithelium with a complete absence of various developmental stages of the parasite was seen (Fig. 8A). Meanwhile, the other portal areas and bile ducts and hepatic parenchyma appeared normal (Fig. 8B).

The hepatocytes regained their original radial shape and appeared almost within the normal histological limit, whereas the hepatic cells were relatively swollen with eosinophilic granular cytoplasm and vesicular nuclei. Scattered individual cell necrosis was also observed. Activation of Kupffer cells associated with mild infiltration of inflammatory cells in hepatic parenchyma was found. Furthermore, hemorrhagic areas and intercellular fibrosis completely disappeared. There were no lesions in hepatic blood vessels and sinusoids (Fig. 8C). The present data proved that using propolis alginate NPs has a valuable coccidiocidal effect and can attenuate *E. stiedae* sporulated oocysts' pathogenicity.

### CD4 + and CD8 + immune cells in the liver

**Fig. 9:**
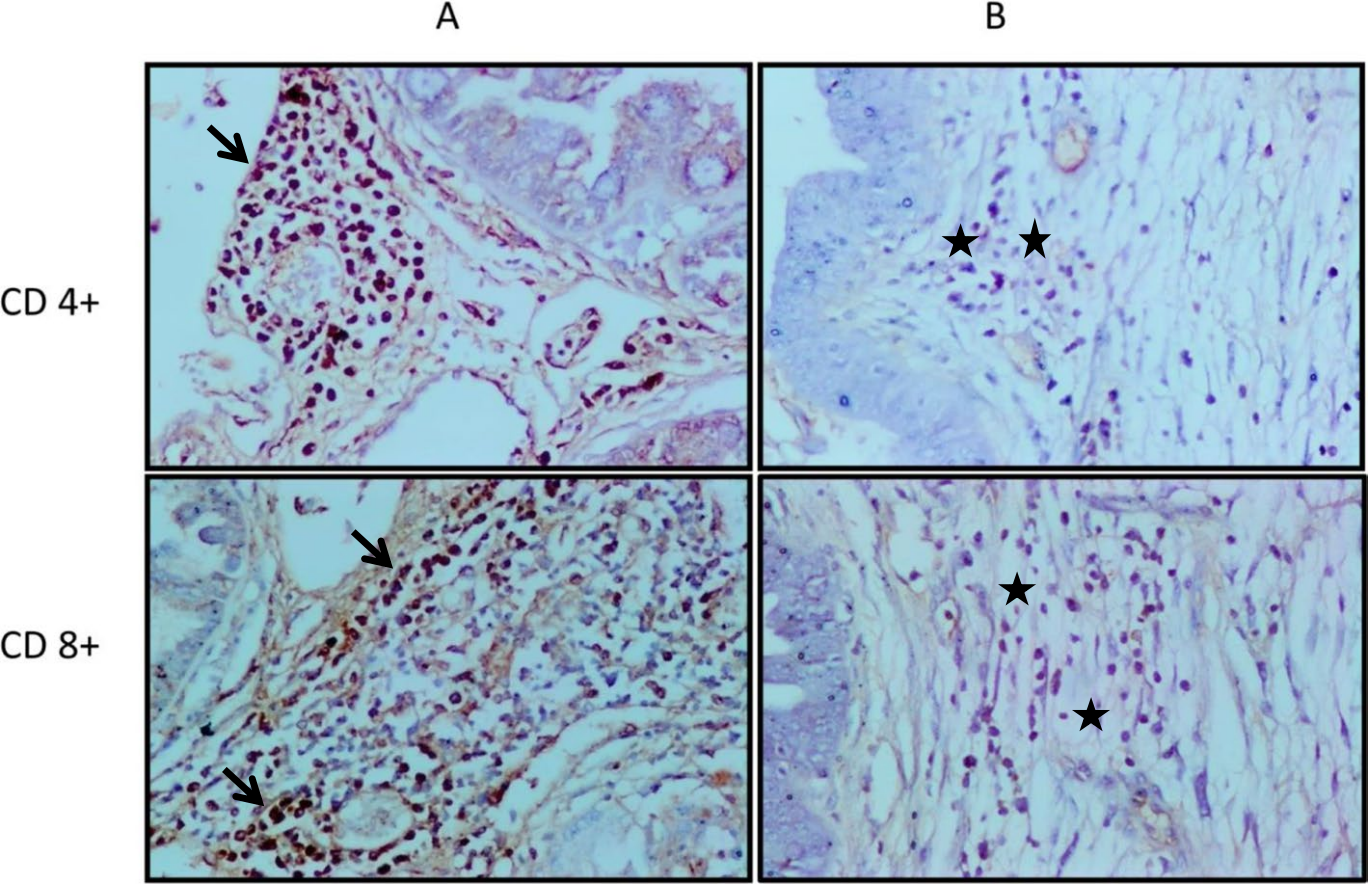
Immunohistochemical staining of CD4 + and CD8 + T lymphocytes that infiltrated in the liver of an infected rabbit with *E. steadie* treated oocyst by alginate propolis NPs (**B**) showing a few numbers of immunostaining CD4 + lymphocytes at the periphery of the bile duct (stars), and showing a few numbers of immunolabelled CD8 + lymphocytes in loose edematous C.T. capsule (stars) surrounding the bile duct. In comparison with an infected rabbit with non-treated sporulated oocysts (control infected group) showed massive aggregations of immunolabelled CD8 + lymphocytes in the portal area (arrow) and massive aggregations of immunolabelled CD4 + lymphocytes (arrow) at the periphery of the bile duct in the portal area (**A**). The dark brown color indicates positive staining. Indirect immunoperoxidase technique (DAB), hematoxylin counter stain X200). Scale bar, 50 µm

Fibrosis and coccidial bile ducts granulomas previously detected with H&E staining in liver tissue sections of the infected control group revealed numerous immunolabelling reactions CD4 + and CD 8 + immune cells along the fibrosis located in the liver of this group, enabling the visualization of the position and presence of these lymphocytes regarding fibrosis formed in the liver (Fig. 9A). Less extent of CD4 + and CD8 + Immunolabelling was noted in liver sections of the group infected with alginate propolis NPs treated oocysts compared with the control infected group (Fig. 9B).

**Fig. 10:**
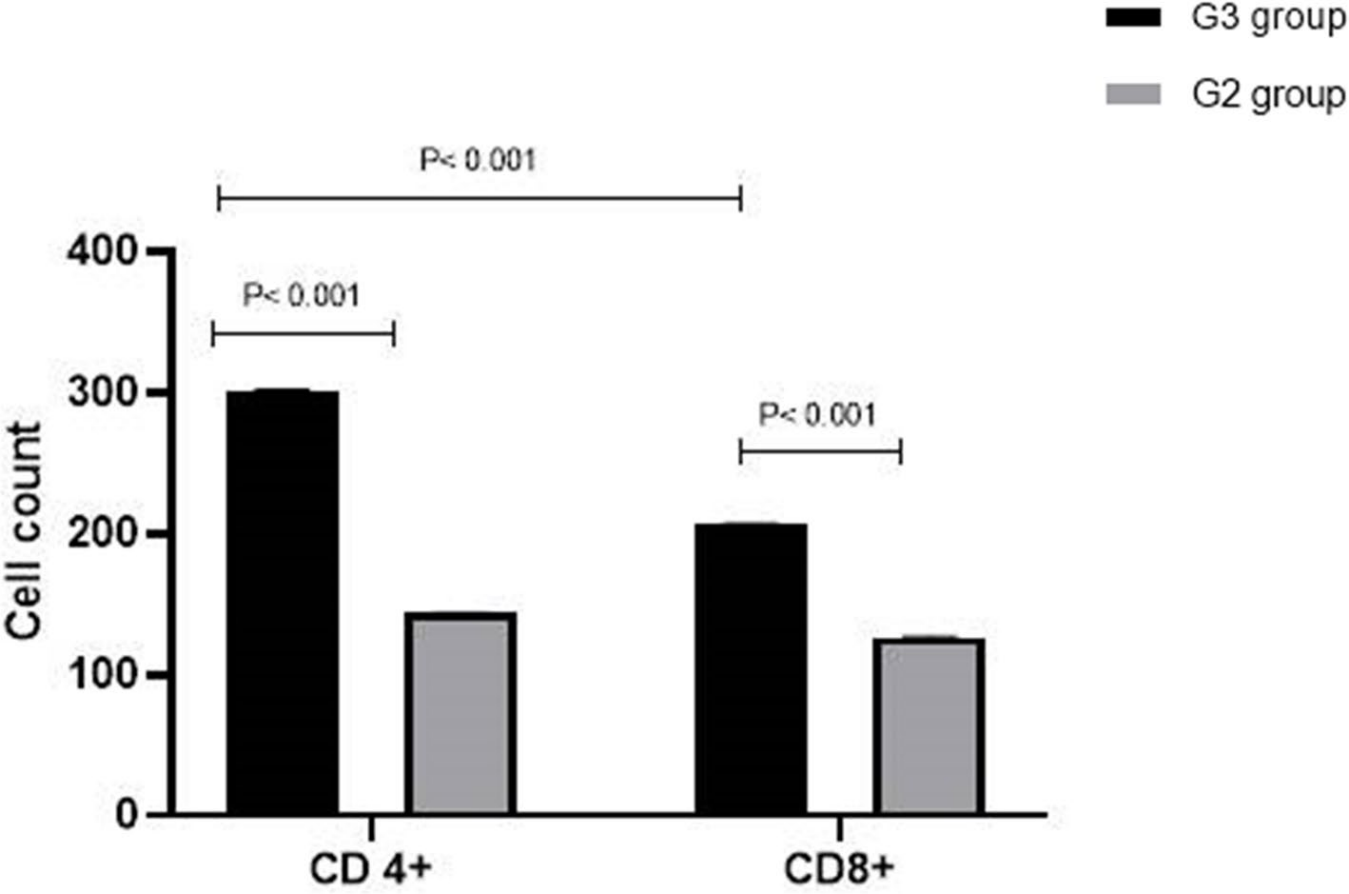
Quantitative study of CD8 + and CD4 + T lymphocytes in the liver section of the group infected with non-treated *E. steadie* sporulated oocysts (G2) and group infected with treated oocysts with alginate propolis NPs (G3). Cells were enumerated as the percentage of positive cells in the field. Results are expressed as mean ± SD. *p*-values were calculated using one-way ANOVA

Quantitative observations of CD4 + and CD8 + were observed in Fig. 10. A comparison of fibrotic and coccidial bile ducts granulomas tissue of the liver of group G2 with group G3 revealed differences. Concerning the number of immunolabeling cells for the CD4 and CD8 antigen (CD4 + , CD8 + T lymphocytes) of group G2 liver cells, a highly significant difference was observed (*p* < 0.001) in comparison with group G3. When comparing the number of CD4 + cells to CD8 + cells in the G2 phase, a significant difference (*p* < 0.001) was seen within the same group. However, in group G3, there is no significant difference in the number of CD4 + and CD8 + cell counts.

## Discussion

Optimizing safety strategies to protect rabbit colonies from hepatic coccidiosis is urgent. Oocyst Inactivation is a better way to prevent the infection. Investigation for new effective anti-*Eimeria* natural products will become more important in controlling these soil and waterborne parasites [60, 84]. In the present study, we synthesized a nanocarrier (Alginate) to combine propolis in a nanosize to increase the surface area, bioavailability, and biological activity and improve propolis tissue permeability to *E. steadie* sporulated oocysts and assess its effect on oocysts infectivity.

The result of TEM revealed the small size of prepared propolis Alg NPs this result may be attributed to that ALg NPs contain anionic carboxylic groups, which cause strong electrostatic repulsion between the particles [85]. The present study using Zeta potential result showed that the value of zeta potential in encapsulated alginate propolis NPs was increased from -60.60 ± 9.10 mV reach to -72.26 ± 6.04 mV. This high value of zeta potential suggests successful encapsulation with high stability. This charge results in strong repulsion forces between the suspended particles and a reduction of aggregations with stable colloidal dispersion. The surface charge has a significant impact on NP cellular absorption. The surface charges of particles, according to Patila et al., can influence how well they adhere to cell membranes [86].

With regard to the clinical features of the treated rabbits, the present study revealed clear improvement in the clinical symptoms of infected rabbits after receiving sporulated oocysts treated with ALg propolis NPs, such as improved food intake, abdominal distension, and dullness, compared to the infected control group, which showed a range of clinical signs—loss of appetite, brown watery diarrhoea, and jaundice—at the second week post inoculation (wpi). The clinical symptoms in the infected control group may be attributed to interference with fat absorption and disturbance in food utilization [87]. These results suggest the ability of ALg propolis NPs to limit *E. stiedae* pathogenicity and reduce inflammatory damage to liver tissue, leading to improvement in the clinical features of the animals.

The estimated numbers of oocyt in faeces demonstrates the severity of the infection and can be used to determine the degree of infectivity of oocyt [88]. In the current study, *E*. *stiedae* oocyst output in faeces was detected in the infected control group at 19 (dpi), and the greatest oocyte count per gram of faeces can be noted between 26 and 29 dpi [5, 8, 89]. The oocyt output in the faeces of rabbits infected with oocyst treated with ALg propolis NPs showed a marked and significant reduction at 25 dpi, and the disappearance of faecal oocyt was observed from the 41st dpi to the end of the experiment; this result may be due to the ability of ALg propolis NPs to reduce the infectivity of sporulated oocysts. Unlike our results, other studies found that different propolis nano-formulations significantly reduced *Eimeria* infestation in rabbits [60]. Interestingly, the great importance of ALg propolis NPs has been described previously [65], as ALg propolis NPs were discovered to have antibacterial synergistic activity against various bacterial strains and as treatment for lumpy skin disease [90].

In the current results, IgG levels started to develop in both groups four days post infection with untreated or treated oocysts. However, their levels were higher throughout the experiment in rabbits infected with untreated oocysts, recording significant differences (*p* ≤ 0.05) 28 days post infection. Specific anti-coccidian antibodies contribute to host defence against infection [91]. Pakandl et al. [92] suggested that cell-mediated immunity, rather than antibody response, is the main adaptive immune response to *Eimeria* infection in rabbits. However, the liver is a lymphoid organ [93] and contains both intrahepatic and migratory T and B cells [94]. The upregulation of IgG in rabbits infected with untreated oocysts, observed in the current study, may reflect an increase in immigration of both T and B cells from the circulation to the liver and the synthesis of specific mRNAs in intrahepatic T and B cells. The significantly low level of IgG in the group infected with treated oocysts may reflect increased emigration of T and B cells from the liver to the circulation and decreased synthesis of these cells with low infectivity of oocysts treated with ALg propolis NPs, which may damage the antigenic profile, resulting in low activation of B cells and, consequently, low levels of IgG.

In the current study, the macroscopic (post-mortem) examination of the rabbits’ livers in the control group infected with *E. stiedae* showed irregular (various shapes and sizes) yellowish-white nodules scattered on the surface as well as moderate hepatomegaly (enlargement in the size of the liver). These findings are similar to others [88, 95, 96]. Such changes could be attributed to severe or extensive proliferation and distention of bile ducts, forming nodules protruding from the liver surface [95], toxic effects of protozoa [88], and fibrosis [5]. Microscopically, the most pronounced characteristics of lesions are severe proliferation (hyperplasia) and high dilatation of bile ducts with extensive biliary epithelium hyperplasia, the presence of the developmental stages of *E. stiedae*, severe degeneration and necrosis of hepatocytes associated with haemorrhages, congestion of hepatic blood vessels, fibrosis, and infiltration of inflammatory mononuclear cells. These histopathological observations agree with those described by others [5, 95–97]. The hyperplasia of the biliary epithelium could be caused by the predilection and proliferation of *E. stiedae* merozoites within the epithelium [95], released toxins, or mechanical irritation induced by a protozoan [5]. In this respect, Cam et al. [97] reported an increase in plasma malondialdehyde (MDA) levels in rabbits experimentally infected with *E. stiedae*. They suggested that the protozoa induced lipid peroxidation by destroying the liver parenchyma and bile duct.

However, the liver of rabbits infected with ALg propolis NPs-treated oocysts showed significant improvement in the histopathological picture, which was more or less similar to those of normal healthy animals; similar results have been reported by Abd El Megid et al. [60], who stated that propolis nanoparticles in protecting rabbits against *E. stiedae* infection could decrease damage to the liver. In contrast, the absence of any protozoal stage in hepatic parenchyma and bile ducts with no or minimal papillary projection of biliary epithelium associated with a decrease in peribiliary fibrosis could be observed. The therapeutic effect of propolis could be attributed to the stimulation of local immune reactions in the elimination of most developmental stages of *Eimeria* [98].

In the presented results, the concentration of IL-12 as proinflammatory cytokines [99] increases in cases of infection with treated oocysts due to the low infectivity of oocysts and a consequently lower grade of inflammation, while in cases of infection with non-treated oocysts with severe infectivity resulted in high inflammation associated with successful infection. At the same time, in the presented data, the observed increase in IL-12, near the levels of IL12 in healthy animals, supports the low infectivity of treated oocysts. It can be proposed as a marker for evaluating the efficacy of ALg propolis NPs in decreasing the infectivity of oocysts. Apart from invading pathogens in the liver, there are activated effector cells that release a variety of mediators, including IL-12. This mediator quickly boosts the local immune response to avoid or reduce the inflammatory stimulus, consequently limiting the inflammation and cleaning up the cellular debris caused by associated tissue damage [100–102].

In the presented study, using immunohistochemical study in the liver of rabbits it was observed that the number of CD4 + and CD8 + T lymphocytes was significantly increased in infected rabbit's liver with *E. stiedae* non-treated oocysts, but increasing CD4 + cells was more conspicuous. Moreover, CD8 + and CD 4 + cells percentage were significantly higher than in those infected with treated oocysts. Our presented results in some regards differ from those of Eladl et al. [8], especially by a high proportion of CD8 + more than CD4 + in the peripheral blood of *E. stiedae* infected rabbit, and this difference may depend on the method of detection and the time of determination of CD4 + and CD8 + as they were determined at 28 dpi in blood by using flow cytometer however in the present study we determine the CD4 at CD8 at 40 dpi using immunohistochemistry. Hermosilla et al. [103] showed that in gut-associated lymph nodes of calves infected with *E. bovis* sacrificed at 35 dpi, there was an increased portion of CD4 + , but not CD8 + , lymphocytes. However, in peripheral blood lymphocytes, the proportions of both CD4 + and CD8 + cells were transiently increased to 12 dpi but decreased to control values at 25 dpi. The importance of mechanisms involving the function of CD4 + T lymphocytes in controlling primary infections with *Eimeria* spp. was demonstrated in mice by Rose et al. [104], in which depletion in CD4 + or CD8 + lymphocytes was detected. Their findings also suggested that CD8 + cells may contribute in some way to expressing resistance to reinfection. Taken together, during primary infection in rodents the number of intraepithelial CD4 + lymphocytes increases, whereas in the reaction to challenge the CD8 + cells are involved. This factor was increased in rabbits infected with the highly immunogenic species *E. stiedae*, indicating the significance of local immune response in eliciting protective immunity against coccidia. A lower percentage of CD4 + and CD8 + T lymphocytes in infected rabbits with treated oocysts suggests that the Alginate-propolis NPs were successful in the pathogenic attenuation of *E. stiedae* sporulated oocysts.

In conclusion, exposure to alginate propolis nanoparticles attenuates *E. stiedae* oocysts as demonstrated in vivo. The Egyptian propolis was encapsulated with Alginate nanoparticles. Infection by *E. stiedae* sporulated oocysts treated with alginate propolis nanoparticles reduces oocysts shedding and consequently low grade of inflammation with remarkable improvement in the histopathological, parasitological, and immunological parameters.

## Data Availability

All datasets of the presented study are available from the corresponding author upon reasonable request.

## Abbreviations

*E. stiedae*: *Eimeria stiedae*
NPs: Nanoparticles
IgG: Immunoglobulin G
FAO: Food and Agriculture Organization
TEM: Transmission Electron Microscope
DLS: Dynamic light scattering
dpi: Day post-infection
wpi: Week post-infection
OPG: Oocysts per gram
ELISA: Enzyme-linked immunosorbent assay
IgG: Immunoglobulin G
IL-12: Interleukin 12
HE: Hematoxylin and eosin
DAB: Diaminobenzidine
ANOVA: Analysis of variance
SD: The standard deviation of the mean
rpm: Revolutions per minute
CaCl2: Calcium chloride
H_2_O_2_: Hydrogen peroxide
FDA: Food and Drugs Administration
