# Correction: Attenuation of pathogenesis of *Eimeria stiedae* sporulated oocysts using Egyptian alginate propolis nanoparticles

**DOI:** 10.1186/s12917-023-03707-z

**Published:** 2023-08-30

**Authors:** Ahmed G. Hegazi, Eman E. El Shanawany, Asmaa S. El-Houssiny, Soad E. Hassan, Hassan M. Desouky, T. M. El-Metenawy, Eman H. Abdel-Rahman

**Affiliations:** 1 Zoonotic Diseases Department, Veterinary Research Institute, National Research Centre, Dokki-Giza, Egypt; 2 Parasitology and Animal Diseases Department, Veterinary Research Institute, National Research Centre, Dokki, Giza, Egypt; 3 Microwave Physics and Dielectric Department, National Research Centre, Dokki-Giza, Egypt; 4 Animal Reproduction and Artificial Insemination Department, National Research Centre, Dokki-Giza, Egypt

**Correction: BMC Vet Res 19, 127 (2023)**

**https://doi.org/10.1186/s12917-023-03689-y**

Following publication of the original article [1], the authors identified the following errors in the in the published version.

- There are errors during the production process in the result section of this article such as Fig. 7 was incorrectly cited and replaced by Fig. 8. Also, citation of Fig. 7F and G in the text was missed so there are paragraph need to be added after the end of paragraph which cited as Fig. 7E. The paragraph that needs to be added is “The hepatic parenchyma showed severe degenerative and necrotic changes of hepatocytes associated with multiple small scattered areas of hemorrhages, in addition to extensive intercellular fibrosis and mononuclear cell infiltration were seen (Fig. 7F). There was centrilobular and perilobular fibrous C.T. proliferation. Moreover, marked dilatation and congestion of the central vein and hepatic sinusoids associated with the hemorrhagic area were observed (Fig. 7G)". The correct figure is given below.

**Fig. 7:**
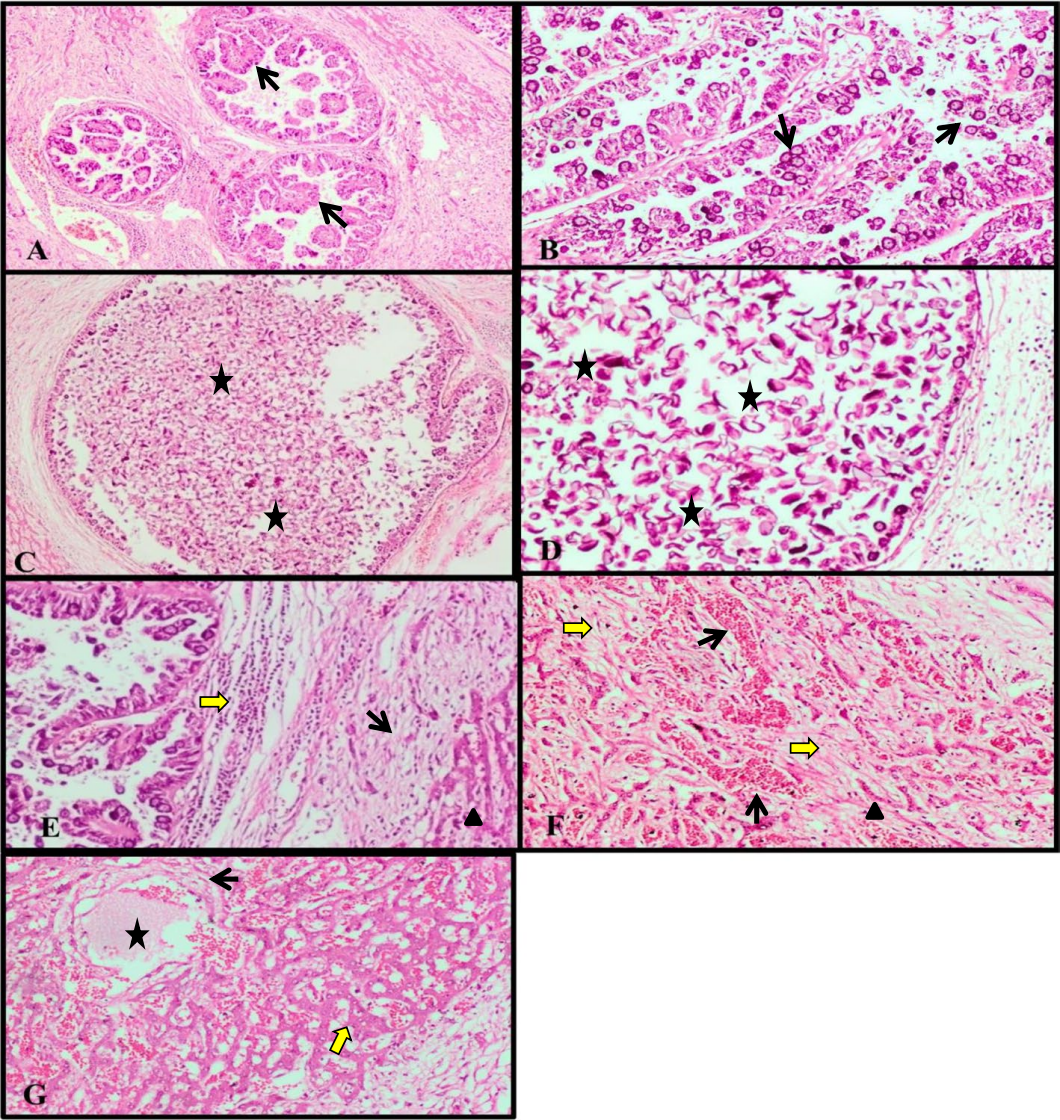
Liver of rabbit infected with *E.steadie* non–treated sporulated oocysts **A** showing proliferation, marked enlargement of bile ducts, extensive hyperplasia of the biliary epithelium forming multiple long papillary projections (arrows) (H&E, X40). **B** Showing invasion of the papillomatous proliferation of biliary epithelium with numerous and various developmental stages of the coccidian parasite (arrows) (H&E, X100). **C** Showing the cystic formation of the bile duct associated with the presence of massive numbers of oval non-sporulated oocysts (stars), and cellular debris within the lumen. (H&E,X100). **D** Higher magnification of figure C (H&E, X200). **E** Showing extensive peribiliary fibrosis (black arrow) associated with infiltration of mononuclear cells (yellow arrow), in addition to necrosis of hepatocytes (arrow head) (H&E, X100). **F** Showing multiple small scattered areas of hemorrhages in hepatic parenchyma associated with fibrosis and severe degenerative and necrotic changes of hepatocytes (yellow arrow) (H&E, X100). **G** Showing high dilatation and congestion of central vein (star) and sinusoids associated with necrosis of endothelial cells lining and centrilobular fibrous C.T. proliferation (black arrow) in addition to necrosis of hepatic cells (yellow arrow) and lymphocytic cell infiltration. (H&E, X100)
